# Editorial: Advanced oral disease therapy: approaches, biotechnology, and bioactive materials, volume II

**DOI:** 10.3389/fbioe.2024.1391548

**Published:** 2024-03-06

**Authors:** Xiaoxuan Zhang, Xianqi Li, Yuan Yin, Jianyun Zhang, Hai Zhang, Xing Wang

**Affiliations:** ^1^ Shanxi Medical University School and Hospital of Stomatology, Taiyuan, China; ^2^ Department of Oral and Maxillofacial Surgery, Matsumoto Dental University, Shiojiri, Japan; ^3^ State Key Laboratory of Military Stomatology, Department of Periodontology, Fourth Military Medical University, Xi’an, China; ^4^ Department of Oral Pathology, Peking University School and Hospital of Stomatology, Beijing, China; ^5^ Department of Restorative Dentistry, University of Washington, Seattle, WA, United States

**Keywords:** biotechnology, bioactive materials, antibacterial, orthodontic, bone defects, diagnosis

## Introduction

In recent years, notable advancements have been made in the research and utilization of biomaterials within the field of dentistry. These developments hold great promise in addressing various dental issues such as defects, bone-related concerns, tumors, infections, and other related conditions. Biomaterials possessing drug delivery capabilities have the potential to modulate cell proliferation and suppress bacterial growth. They play a crucial role in the restoration of defects in hard tissue like teeth and bones, as well as in the management of infected wounds. Simultaneously, investigations into disease mechanisms offer the potential for the development of novel therapeutic interventions.

## Antibacterial property of dental materials

The presence of bacteria in the oral cavity can lead to dental caries and peri-implantitis. The antibacterial properties of biomaterials are crucial.

Efficiently eliminating dental plaque from implant surfaces has emerged as a critical concern that requires immediate attention in the realm of disease prevention and treatment around implants. With the advancement of materials and pharmacology, extensive research has been undertaken to improve the antibacterial efficacy of implants. This includes the application of antibacterial coatings on implant surfaces, adjusting the surface morphology of implants, and developing new implant materials. The research on surface modification of implants and enhancing their antibacterial properties is reviewed by Zhai et al., providing theoretical basis for the prevention of diseases around implants in the future. In the samples of periimplantitis lesions, there was a significant increase in M1 macrophages to pro-inflammatory. In order to better understand the pathogenesis of periimplantitis, the review by Li et al. summarizes research related to macrophage polarization in periimplantitis, and proposes potential immunomodulatory treatment options for periimplantitis. Li et al. designed a novel biomimetic nano-morphology coating on the surface of titanium-based implants with antibacterial copper by combining plasma electrolytic oxidation and hydrothermal treatment. The biological interaction between the coating and stem cells promotes cell adhesion, diffusion, and proliferation during the initial attachment process, and this new coating reduces the activity of mixed bacteria (*Fusobacterium* nucleatum and Porphyromonas gingivalis).

In terms of tooth hard tissue infectious diseases, Qu et al. reviewed the anti-caries application of chitosan, and focused on classifying and summarizing the characteristics of chitosan as a caries-prevention material, including its antibacterial effect, biomimetic mineralization, and the delivery ability of caries-prevention drugs and vaccines.

The insufficient corrosion resistance and antibacterial performance of fixed orthodontic devices lead to enamel demineralization. Chen et al. constructed a uniform and dense polydopamine graphene oxide nanocoating on nickel-titanium (NiTi) archwires by self-assembly, reducing Ni dissolution in NiTi alloys and exhibiting antibacterial activity against *streptococcus* mutans.

In the case of a skin infection resulting from the presence of biofilm, Li et al. developed a hydrogel containing the active ingredient berberine hydrochloride liposomes to destroy the biofilm, thus accelerating the healing of infected wounds in mice.

## Biomaterial for regeneration of bone defects

Biomaterial must adhere to stringent criteria for the reconstruction of bone defects in the craniomaxillofacial region. It necessitates adequate support and surface porosity to facilitate cell attachment and vascular growth. Additionally, the material should possess bone induction and conduction capabilities to encourage the crawl and growth of adjacent bone tissue. Metal materials have become important bone scaffold materials with advantages of support and plasticity. Zhang et al. used a 3D printing technology to produce porous tantalum scaffolds, and then constructed the nano-morphology of the scaffold surface through alkali heat treatment with NaOH solution and hydrothermal treatment in deionized water. The tantalum scaffold has promoted the proliferation and osteogenic differentiation of BMSCs and accelerated the formation of new bone in the mandibular defect angle of rabbits, achieving rapid bone integration. Compared with Ti_6_Al_4_V, tantalum has better mechanical properties, osteogenic ability, and microstructure, becoming a potential alternative material for bone repair. Large-area bone defects are often accompanied by loss of surrounding soft tissue. 3D printing-assisted fibula osteoseptocutaneous flap combined with antagonistic thick flaps is used to repair a wide range of composite defects. Xu et al. evaluated the repair capacity and patient satisfaction of this treatment, elaborated on the advantages and challenges of the method, and provided a basis for the selection of clinical surgical treatment.

## Research for orthodontic aligners

Orthodontic clear aligners have gained popularity among the general population because of their aesthetic appeal; however, there is a lack of evidence regarding the mechanical performance of transparent braces in the oral cavity. Cintora-López et al. investigated the effects of temperature and load on the deformation of aligners. Mechanoluminescent materials provide the possibility for visualizing stress changes. A bite splint was prepared by mixing phosphors with polydimethylsiloxane, which can establish the relationship between mechanical force and light signal. Zhang et al. evaluated the biological safety of bite splints mixed with BaSi_2_O_2_N_2_ phosphors in order to explore the possibility for clinical application.

## Diagnosis and treatment of oral tumors and immune diseases

Early detection of various oral diseases, including tumors, is advantageous for predicting outcomes. Currently, an accurate diagnosis depends on tissue biopsy, a highly invasive surgical procedure with limited timeliness. Extracellular vesicles participate in intercellular communication, promote disease progression, and reflect the location and status of lesions. The review by Zhang et al. discusses the involvement of extracellular vesicles in the diagnosis, development, and treatment of oral squamous cell carcinoma (OSCC), providing new insights for the diagnosis and treatment of OSCC. Sjogren’s syndrome is an autoimmune disease characterized by the destruction of salivary glands (SG), leading to loss of secretion function and the presence of focal lymphocyte infiltration in the SG. Hasegawa et al. used effective monocytes to inhibit lymphocyte infiltration and alleviate SG damage in Sjögren syndrome.

## Conclusion

With the advancement of technology, the utilization of biomaterials in oral diagnosis and treatment is progressively expanding, offering a wider range of functionalities. Various tools and materials, such as scaffolds designed with osteogenic properties, aligners capable of detecting mechanical changes, dental filling materials for treating caries, and substances with antibacterial properties, are utilized to address issues in the intricate oral microenvironment. The ongoing advancement of research into the pathogenesis of oral diseases will enhance the precise and prompt diagnosis and treatment of oral conditions.

